# Accumulation of isolevuglandin-modified protein in normal and fibrotic lung

**DOI:** 10.1038/srep24919

**Published:** 2016-04-27

**Authors:** Stacey Mont, Sean S. Davies, L. Jackson Roberts second, Raymond L. Mernaugh, W. Hayes McDonald, Brahm H. Segal, William Zackert, Jonathan A. Kropski, Timothy S. Blackwell, Konjeti R. Sekhar, James J. Galligan, Pierre P. Massion, Lawrence J. Marnett, Elizabeth L. Travis, Michael L. Freeman

**Affiliations:** 1 Department of Cancer Biology, Vanderbilt University Medical Center, Nashville, TN 37240, USA; 2 Department of Radiation Oncology, Vanderbilt University Medical Center, Nashville, TN 37240, USA; 3 Division of Clinical Pharmacology, Department of Pharmacology, Vanderbilt University Medical Center, Nashville, TN 37240, USA; 4 Department of Biochemistry, Vanderbilt University Medical Center, Nashville, TN 37240, USA; 5 Proteomics Laboratory and Mass Spectrometry Research Center, Vanderbilt University Medical Center, Nashville, TN 37240, USA; 6 Department of Medicine, Department of Immunology, Roswell Park Cancer Institute, and University at Buffalo Jacobs School of Medicine and Biomedical Sciences, Buffalo, NY, 14263, USA; 7 Division of Pulmonary & Critical Care, Department of Medicine, Vanderbilt University Medical Center, Nashville, TN 37240, USA; 8 A.B. Hancock Jr. Memorial Laboratory for Cancer Research, Vanderbilt Institute of Chemical Biology, Vanderbilt-Ingram Cancer Center, Vanderbilt University Medical Center, Nashville, TN 37240, USA; 9 Department of Experimental Radiation Oncology, Division of Radiation Oncology, The University of Texas MD Anderson Cancer Center, Houston, TX 77230, USA.

## Abstract

Protein lysine modification by γ-ketoaldehyde isomers derived from arachidonic acid, termed isolevuglandins (IsoLGs), is emerging as a mechanistic link between pathogenic reactive oxygen species and disease progression. However, the questions of whether covalent modification of proteins by IsoLGs are subject to genetic regulation and the identity of IsoLG-modified proteins remain unclear. Herein we show that Nrf2 and Nox2 are key regulators of IsoLG modification in pulmonary tissue and report on the identity of proteins analyzed by LC-MS following immunoaffinity purification of IsoLG-modified proteins. Gene ontology analysis revealed that proteins in numerous cellular pathways are susceptible to IsoLG modification. Although cells tolerate basal levels of modification, exceeding them induces apoptosis. We found prominent modification in a murine model of radiation-induced pulmonary fibrosis and in idiopathic pulmonary fibrosis, two diseases considered to be promoted by gene-regulated oxidant stress. Based on these results we hypothesize that IsoLG modification is a hitherto unrecognized sequelae that contributes to radiation-induced pulmonary injury and IPF.

Isolevuglandins (IsoLGs) are a family of eight γ-ketoaldehyde regioisomers formed by non-enzymatic rearrangement of endoperoxide intermediates produced when arachidonic acid or its phospholipid esters undergo enzymatic or free radical mediated cyclooxygenation^1^,^2^. The endoperoxide intermediate produced enzymatically by cyclooxygenases (prostaglandin H_2_) can form two of the eight potential regioisomers, while the endoperoxide intermediates produced by free radicals can form all of the eight potential regioisomers^1^,^2^. Emerging research indicates that modification of proteins by IsoLGs can contribute to the pathogenesis of diseases driven by oxidative stress^3^,^4^,^5^,^6^. IsoLG modification can be a proteotoxic event that inhibits a number of different processes such as macrophage-mediated degradation of LDL^3^, sodium channel function^6^ and proteasome function^7^. It can also be immunogenic^8^. Currently, however, there is a lack of knowledge concerning the identity of susceptible proteins and whether IsoLG formation and accumulation of modified proteins is genetically regulated. Although IsoLG modification can be cytotoxic, the underlying molecular subroutine that drives cell death has not been identified.

The primary function of NADPH oxidases, a superoxide-generating family of enzymes consisting of 7 NOX members (NOX1-NOX5, DUOX1, and DUOX2) is the generation of reactive oxygen species (ROS)^9^. While generation of ROS by NOX can directly lead to IsoLG formation via the free radical pathway, NOX activation also leads to cyclooxygenase activation^10^,^11^ and thus potentially to additional formation of IsoLGs via the cyclooxygenase pathway. The prototypical Nox2 enzyme is a multicomponent complex that generates superoxide and consists of a membrane bound flavocytochrome-based oxidase, gp91, heterodimerized to p22 ^phox^. Activation requires assembly of cytosolic-based p40 ^phox^, p47 ^phox^, and p67 ^phox^, as well as the small GTPase Rac^12^. Nox2 is expressed in phagocytic cells, vascular tissue and in most pulmonary cells^13^ and its activity is critically dependent upon the Nox adaptor protein p47 ^phox^^9^ encoded by *NCF1*. While Nox2 plays a critical role in antimicrobial host defense it also can be injurious. Herein we report that Nox2 promotes pulmonary IsoLG modification.

Nrf2-dependent gene expression represents a crucial pathway for suppressing a ROS challenge. The transcription factor Nrf2, encoded by *Nfe2l2*, is a highly conserved member of the cap ‘n’ collar (CnC) family of basic leucine zipper transcription factors^14^, promotes antioxidant gene expression (eg, HO1, SOD1, SOD2, GCLC, GCLM, and CAT^15^,^16^) and is essential for maintaining ROS homeostasis^17^. Consistent with this concept, EPR imaging has shown that tissue ROS is increased in the Nrf2 null mouse^18^. In addition, Nox2 stimulates Nrf2 activation in lung tissue during inflammation in mice, and Nrf2 limits injury^19^. We found that IsoLG-modified protein was significantly increased in the lungs of Nrf2 deficient mice, thus identifying Nrf2 gene expression as a critical nexus for suppressing IsoLG modification of protein.

Loss of Nrf2 activity^20^,^21^ and elevated NAPDH oxidase-mediated ROS^22^,^23^ is noted in murine models of radiation-induced pulmonary fibrosis as disease progresses. This knowledge leads to the prediction that IsoLG modified protein should be a prominent feature in this disease. First, we show that exposure to ionizing radiation, a stress that produces various types of ROS^24^, induced IsoLG modification in a cell culture model. Then using a proteomic approach we identified and compared proteins that have the potential to be modified in irradiated and non-irradiated cells. Viability studies demonstrated that apoptosis results when a threshold of IsoLG modification is exceeded. Next, we assessed and found high levels of modification in irradiated murine lung compared to sham irradiated tissue.

Abrogation of Nrf2 expression compounded by elevated NAPDH oxidase activity also characterizes human idiopathic pulmonary fibrosis (IPF)^25^,^26^,^27^. Therefore we assessed adduction in IPF and human donor lung tissue. High levels of IsoLG-modified protein were observed in pulmonary tissue from IPF patients but not in control tissue, consistent with the knowledge that oxidant stress is central to the progression of IPF. The proteomic analysis identified collagen 1 α 1 as a protein highly susceptible to modification and this was confirmed in IPF tissue using immunofluorescence confocal microscopy. Strikingly, modified collagen 1α1 was found to be resistant to MMP-1-mediated degradation, suggesting that IsoLG modification can impact tissue remodeling.

## Results

### IsoLG-modified proteins are present in the lungs of unstressed, healthy mice

**Figure 1:**
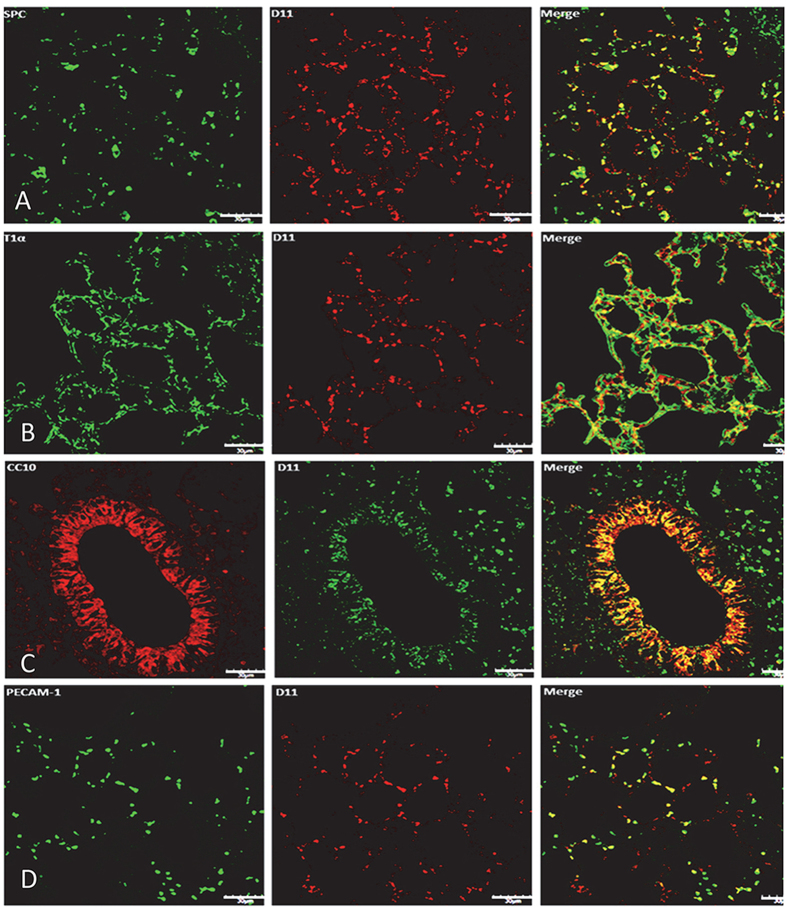
IsoLG-modified proteins are present within several lung cell populations in unstressed mice. Mouse lung tissue sections were immunostained with the following: (**A**) anti-SPC for Type 2 alveolar cells (Alexa 647 secondary, green false color), D11 ScFv (Rhodamine Red secondary); (**B**) anti T1α for Type 1 alveolar cells (Alexa 647 secondary, green false color), D11 ScFv (Rhodamine Red, secondary); (**C**) anti-CC10 for Club cells (Rhodamine Red secondary), D11 ScFv (Alexa 647 secondary, green false color); (**D**) anti-PECAM-1 for endothelial cells (Alexa 647 secondary, green false color), D11 ScFv (Rhodamine Red secondary). Images were acquired using confocal microscopy (60× magnification). The white bar represents 30 μm.

We determined the basal level of IsoLG-modified protein in murine lung obtained from non-diseased C57BL/6J mice maintained under stress-free conditions and continuous access to water, food and regular 12 hr light cycles. Lung tissue sections underwent dual-immunofluorescence staining for IsoLG-modified proteins and Type 2 alveolar epithelial cells (SPC, Fig. 1A), Type 1 alveolar epithelial cells (T1α, Fig. 1B), Club cells (CC10, Fig. 1C), or endothelial cells (PECAM-1, Fig. 1D). IsoLG-modified proteins were identified using the well characterized single chain antibody D11 ScFv, which recognizes peptides and proteins modified by IsoLG isomers that can arise from both the free radical and cyclooxygenase mediated pathways^4^,^28^,^29^,^30^. We observed IsoLG-modified protein in each cell type, suggesting that the burden of IsoLG-modified protein is a byproduct of normal lung metabolism.

### NADPH oxidase promotes, while Nrf2 suppresses, the generation of IsoLG-modified protein

**Figure 2:**
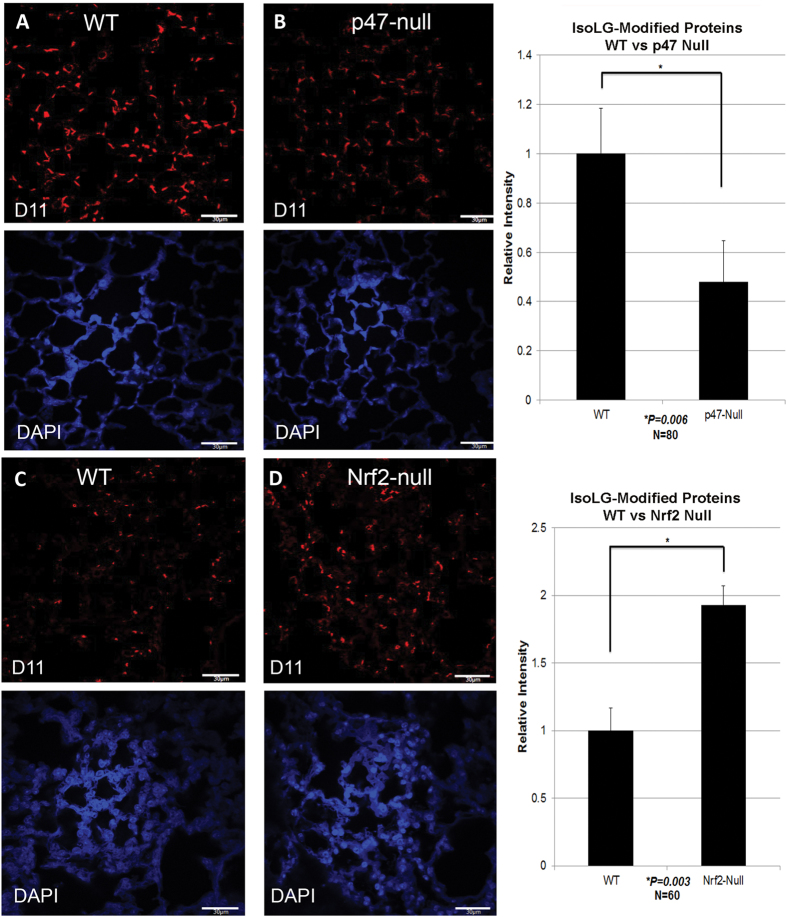
IsoLG-modified protein in formalin fixed paraffin embedded pulmonary tissue from age matched wild type (A and C, N = 6), p47^phox^ null (B, N = 3), or Nrf2 null (D, N = 3) mice. Images were acquired using confocal microscopy (60× magnification). Quantification of D11 ScFv immunofluorescence, corrected for DAPI staining is shown on the right for p47 ^phox^ null vs wild type (N = 80 random fields) and for Nrf2 null vs wild type (N = 60 random fields). White bars represents 30 μm.

p47 ^phox^ is an activating subunit of Nox2, a major source of cellular superoxide. Quantification of D11 immunofluorescent/DAPI staining in pulmonary tissue obtained from C57BL/6J wild type (Fig. 2A) and age-matched p47 ^phox^ null mice (Fig. 2B) demonstrate that loss of NADPH oxidase activity suppressed adduction by 55% (*P* = 0.006, Fig. 2). These data demonstrate that NADPH oxidase activity is a major source for the generation of IsoLG-modified protein.

The Nrf2-null mouse represents a well characterized model for investigating ROS interactions. Pulmonary tissue was obtained from Nrf2-null and age-matched wild type C57BL/6J mice. Quantification of D11 immunofluorescent/DAPI staining in pulmonary tissue obtained from wild type (Fig. 2C) and Nrf2-null mice (Fig. 2D) revealed that loss of Nrf2 resulted in a 2-fold increase in IsoLG-modified protein (*P* = 0.003, Fig. 2). Taken together, these data show that genes regulating oxidative stress can substantially alter adduction in pulmonary tissue.

### IsoLG-modified proteins are present in human lung

Having shown that IsoLG-modified proteins are present in mouse lung we asked whether human lung also exhibited a basal level of IsoLG modification. Formalin-fixed paraffin-embedded (FFPE) pulmonary tissue obtained from 2 control organ donors (Subjects 5 and 6, Supplementary Table 1) underwent IHC staining with the D11 antibody, counterstained with methyl green and imaged by wide field microscopy (Supplementary Fig. 1). Very little D11 immunoreactivity was noted. Next, confocal microscopy was used to image FFPE tissue immunostained with D11 and counter stained using DAPI. The confocal images illustrate the presence of a low level of IsoLG-modified protein in human lung tissue (Supplementary Fig. 1), confirming the observations of others^30^.

### Low expression of NRF2 in human lung

Given that loss of Nrf2 can potentiate formation of IsoLG-modified protein in murine lung we investigated NRF2 expression in human lung. De-identified fine-needle bronchial biopsies, obtained from 40 subjects, were used to quantify the expression of *NFE2L2* mRNA by qRT-PCR (Supplementary Fig. 2A). Subject characteristics are shown in Supplementary Table 2. The small amount of tissue present in the biopsy samples precluded analysis by immunoblotting. The median value was 0.0068 ng *NFE2L2* mRNA per 12 ng of total RNA. Strikingly, we found a 50-fold difference in the normalized expression of *NFE2L2* mRNA (*P* < 0.05).

NQO1 is a validated NRF2 target gene^31^. We assessed the expression of *NQO1* mRNA in a subset of individuals and found a statistically significant correlation between expression of *NFE2L2* mRNA and expression of NQO1 mRNA (Pearson’s correlation coefficient = 0.80, *P* = 0.01, Supplementary Fig. 3). With the caveat of the small sample size, these results suggest the possibility that subsets of individuals may be deficient in NRF2 expression, increasing their potential susceptibility to oxidative stress and resulting protein modification by IsoLGs.

### LC-MS analysis of immuno-affinity purified IsoLG-modified proteins

**Figure 3:**
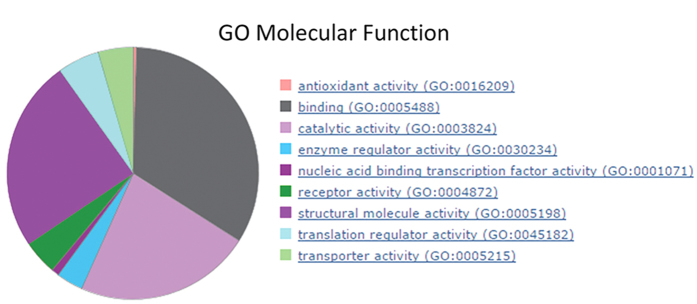
PANTHER analysis of endogenous IsoLG-modified proteins. Protein lysates from mouse 3B11 endothelial cells were immunoprecipitated with D11 ScFv, subjected to LC/MS/MS and then analyzed using the PANTHER software.

Currently, there is little knowledge concerning the identity of proteins that are susceptible to IsoLG modification. We chose to investigate the identity of proteins adducted endogenously using endothelial cells due to their well characterized and robust NADPH oxidase activity^32^. IsoLG-modified proteins from 3B11 endothelial cells were immuno-affinity purified using the D11 antibody. Proteins were subjected to LC/MS/MS analysis. Analysis of 3 independent experiments identified 162 proteins. A PANTHER network and pathway analysis^33^ of Molecular Function was performed (Fig. 3 and Supplementary Table 3, tab immunoprecipitated protein) revealing that proteins susceptible to adduction are not restricted to an organelle or class of proteins, but can be classified into several distinct cellular pathways (Fig. 3).

**Figure 4:**
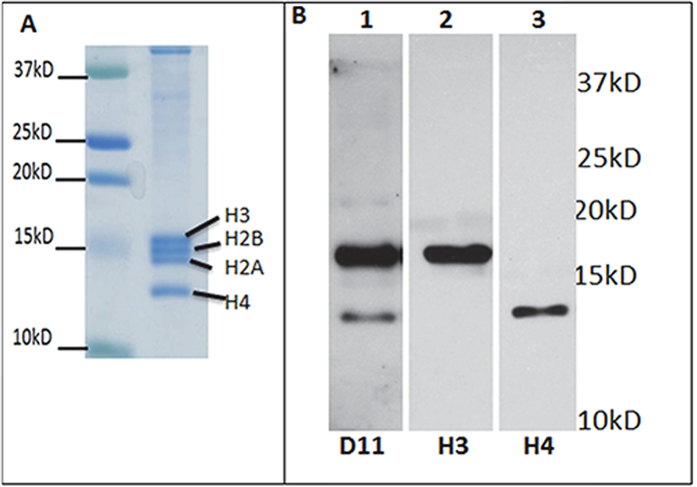
Identification of IsoLG-modified histone-H3 and -H4 in mouse lung. (**A**) Coomassie staining of histones isolated from mouse lung; (**B**) Immunoblot of isolated histones using D11 ScFv (lane 1), Histone H3 (lane 2) or H4 (lane 3) antibody.

Histones have been shown to be susceptible to modification by reactive lipid electrophiles, including IsoLGs (specifically levuglandin E_2_) and 4-oxononenal, resulting in stable lysine adducts^34^,^35^. Our analysis demonstrated the presence of histone H4, H1.3, H1.4, H2A, H2B, and H3.3 peptide profiles detected by mass spectrometry following D11-mediated purification (Supplementary Table 3, tab Binding). We measured IsoLG-histone modification by isolating chromatin from mouse whole lung homogenates^35^ (Fig. 4A). Immunoblotting of chromatin with D11 confirmed the modification of histones H3 and H4 in mouse lung tissue (Fig. 4B).

### Ionizing radiation promotes the formation of IsoLG-modified proteins

Seventy percent of X- and gamma-ray photons traversing a tissue interact with water molecules that rapidly decompose into hydroxyl radicals (·OH), hydrogen radicals (·H), hydrogen peroxide, superoxide, and solvated electrons^36^. We determined if irradiation could generate IsoLG-modified protein in a cell culture model. Although alveolar epithelial cells and fibroblasts are key to the pathogenesis of radiation–induced pulmonary fibrosis^37^, their response may be a consequence to radiation-induced pulmonary microvascular injury, a prominent sequelae that manifests hours after irradiation of human, dog, rat, and mouse lung^38^,^39^,^40^,^41^,^42^,^43^. Thus, we again chose an approach that utilized endothelial cells. Human Microvascular Endothelial Cells (HMVECs) were exposed to 5 Gy of Cesium-137 γ-rays. Quantification of immunofluorescence D11 staining for IsoLG-modified proteins show a 2-fold increase 24 hours after irradiation (*P* = 0.001, Supplementary Fig. 4A,B). Because hydrogen peroxide (H_2_O_2_) is formed following photon irradiation, we determined if H_2_O_2_ would induce IsoLG-modification. HMVECs were exposed to 150 μM H_2_O_2_ for 1 hr at 37 °C and then allowed to recover for 24 hrs prior to immunostaining with D11. As shown in Supplementary Fig. 4A,B, H_2_O_2_ treatment caused a 4.4 fold increase in IsoLG-modified protein (*P* = 8.4 10^−7^).

### MS-MS analysis of immuno-affinity IsoLG-modified proteins from irradiated 3B11 endothelial cells

IsoLG-modified proteins were immuno-affinity purified from 3B11 cells 24 hrs after administering 5 Gy. Isolated proteins were again subjected to LC/MS/MS (Supplementary Table 4). Ninety four percent of the proteins identified in the irradiated samples were also found in sham treated samples. Proteins specific to the irradiated samples are noted in red font, Supplementary Table 4. Based on the observations that there was a 2 fold increase in D11 immunoreactivity in irradiated cells compared to sham (Supplementary Fig. 4A) we hypothesize that cells contain a ‘pool’ of IsoLG-modification susceptible proteins, with the fraction of proteins modified within the pool increasing as oxidant stress increases.

### IsoLGs are cytotoxic and promote apoptosis

We assessed the consequence of increasing the cellular burden of modified protein by exposing 3B11 and HMVECs to a bolus of a synthetic IsoLG isomer (15-E_2_-IsoLG) in PBS (1 μM/1 hr). 15-E_2_-IsoLG is one of the IsoLG regioisomers that can be produced by both the free radical pathway and the cyclooxygenase pathway (i.e. levuglandin E_2_) and its synthesis was described in^44^. Apoptosis induced by 15-E_2_-IsoLG was then assessed 16 hrs later by quantifying Annexin V positive/propidium iodine negative staining (Supplementary Fig. 5). Exposure to 15-E_2_-IsoLG (EC_50_ = 1 μM) produced statistically significant increases in apoptosis, as measured by Annexin V positive/propidium iodine negative staining in both cell types.

As stated above alveolar type II cells are a key component to development of pulmonary fibrosis. Therefore it was of interest to determine if type II cells were susceptible to IsoLG-mediated cytotoxicity. SV40 transformed mouse MLE12 alveolar type II cells were exposed to various concentrations of 15-E_2_-IsoLG for 1 hr in PBS. Cytotoxicity, as measured by a MTT assay, was quantified 16 hrs later. As shown in Supplementary Fig. 5B, 15-E_2_-IsoLG was cytotoxic.

### Accumulation of IsoLG-modified protein in radiation-induced lung injury and in IPF

**Figure 5:**
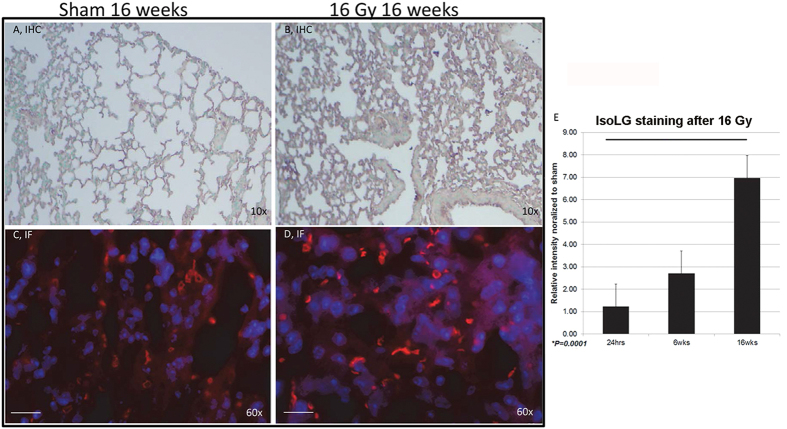
Ionizing radiation can induce the formation of IsoLG-modified proteins *in vivo*. Mouse pulmonary tissue was obtained 16 weeks after exposure to sham irradiation (**A**,**C**) or 16 Gy (**B**,**D**). (**A**,**B**) IHC D11 ScFv immunostaining, counterstained with methyl green. (**C**,**D**) D11 ScFv immunofluorescence (IF) counterstained with DAPI. Random fields are shown. (**E**) Mean (±SD) immunofluorescence staining was measured at 60× magnification by wide-field microscopy (40 fields per time point obtained from 12 mice). White bar represents 30 μm.

Suppression of Nrf2 and elevation of NADPH oxidase-mediated oxidant stress as disease progresses^21^,^25^,^26^ are two prominent features of radiation-induced lung injury and human IPF^22^,^23^,^26^,^27^,^45^. Therefore, we asked if IsoLG modification also accompanies radiation injury. Wildtype C57BL/6J mice were administered a thoracic dose of 16 Gy, a dose that induces fibrotic lesions within 16 weeks^46^. Strikingly, we found that 16 Gy significantly increased the degree of D11-mediated immunofluorescence 6 and 16 weeks after irradiation (Fig. 5, panels C-E). Six weeks after 16 Gy there was a nearly 3-fold increase, which increased a further 2.3 fold 16 weeks after treatment as compared to sham control (Fig. 5E). Our findings correlate the onset and persistence of pulmonary fibrosis^46^ with the formation and accumulation of IsoLG-modified proteins.

**Figure 6:**
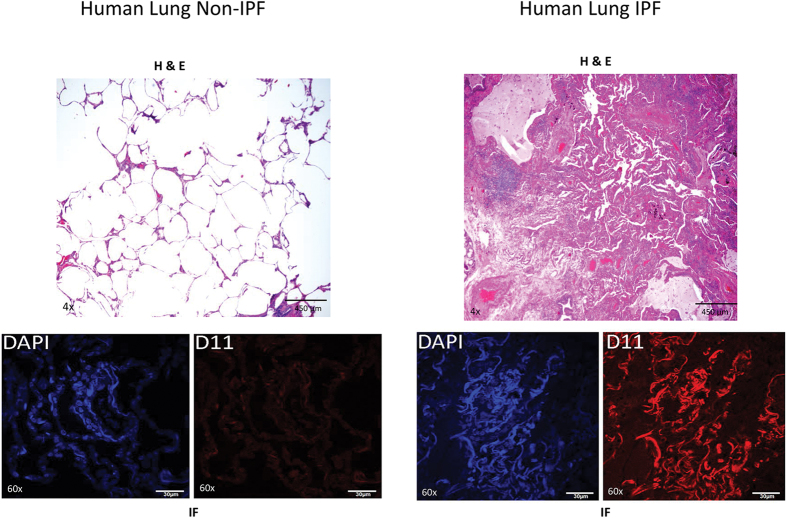
Human lung tissue sections obtained from non-IPF organ donors (N = 3) and IPF patients (N = 3). FFPE sections underwent H&E staining or subjected to IHC staining with D11 ScFv and counterstained with methyl green. Sections were imaged using wide field microscopy. Other sections were imaged by confocal microscopy following immunofluorescent staining with D11 ScFv and counter staining with DAPI. White bars in IF sections represent 30 μm.

Human lung tissue was obtained from 3 organ donor individuals and 3 IPF patients who underwent lung transplant. Fibrotic tissue present in IPF samples is apparent by H&E staining (Fig. 6). Comparison of D11 immunofluorescence staining among the samples revealed a 4-fold increase in IsoLG-modified proteins in IPF tissue compared to organ donor controls (*P* = 5.8 × 10^−41^, N = 100 fields, Fig. 6). Inspection of IHC staining using the D11 antibody and counterstained with methyl green indicated the presence of IsoLG-modified protein in single cells (Supplementary Fig. 6A–C) and in multi-cellular lesions (eg Supplementary Fig. 6G).

IPF dense fibrotic tissue was co-immunostained for collagen 1α1 and IsoLG-modified protein. As illustrated in Supplementary Fig. 7 IsoLG-modified protein co-localized with collagen 1α1 in IPF tissue, consistent with the results obtained from the MS analysis (Supplementary Table 3, tab structural (GO: 0005198)).

We next addressed whether IsoLG-modified collagen 1α1 is resistant to MMP1-mediated degradation. 15-E_2_-IsoLG modification of collagen 1α1 impaired its degradation by MMP1 in a concentration-dependent manner (Supplementary Fig. 8). This finding is consistent with the results reported by Davies *et al*. who have shown that IsoLG modification of protein impedes its proteolysis^47^.

## Discussion

Although it is well established that oxidative stress promotes progression of many diseases, the underlying mechanisms are not well understood. As shown by emerging research, IsoLGs are a proteotoxic and immunogenic stress that links ROS to progression of disease^3^,^4^,^5^,^6^,^29^. Our novel results indicate that IsoLG-modified protein has a significant presence in radiation-induced pulmonary injury and IPF. We demonstrate for the first time that genetic regulators of oxidative stress play a critical role in adduction. Furthermore, our findings that IsoLG is proapoptotic and modifies collagen in IPF tissue and that modified collagen is resistant to proteolysis by MMP1 point to IsoLG as one plausible mechanism driving chronic pulmonary injury and fibrosis.

The presence of IsoLG-modified protein in alveolar epithelium and endothelium, as well as in bronchiolar epithelium of heathy mice indicates that cells can tolerate a basal level of protein adduction. We have shown that a cell’s IsoLG load is directly related to its oxidant burden. Nox2 catalyzes electron transfer from NADPH to molecular oxygen, generating superoxide, which can dismutate into H_2_O_2_^48^ and is expressed in most pulmonary cells, including alveolar macrophages, and dendritic cells^13^. The importance of Nox2 as a major cellular source of oxidant stress driving IsoLG modification in the lung is underscored by the reduction in D11 immunostaining in p47 ^phox^ deficient mice. Although the free radical pathway is a likely route of IsoLG formation under these conditions, the cyclooxygenase pathway of IsoLG formation may also contribute as formation of ROS by Nox can upregulate COX2 activity^10^,^11^. Nrf2, which is ubiquitously expressed, promotes antioxidant gene expression (eg, SOD1, SOD2, and CAT^49^) and is critical for maintaining ROS homeostasis^17^. We found that pulmonary tissue from Nrf2-null mice exhibited significantly increased levels of IsoLG-modified protein, thus identifying Nrf2 gene expression as important for the suppression of modification. The variance in pulmonary NRF2 expression among a subset of individuals sampled in our study suggests that the risk for an oxidative burden and formation of IsoLG-modified protein could be exacerbated by the onset of disease in those with endogenously low levels of NRF2.

Immuno-isolation of IsoLG-modified protein followed by MS/MS analysis allowed us to identify over 160 protein targets. Although irradiation produced significantly more modification, the majority of the proteins were the same for irradiated and unirradiated cells. One interpretation for these results is that cells contain a pool of susceptible protein, so that increasing the oxidant stress primarily increases the fraction of each susceptible protein within the pool that is modified. PANTHER network analysis revealed that proteins from multiple organelles and pathways are susceptible to be modified.

IsoLG protein-adduction can be a proteotoxic event, a consequence of protein misfolding, aggregation, and/or crosslinking^34^,^50^,^51^,^52^,^53^,^54^. IsoLG-adducted proteins can be immunogenic^8^ and activate T cells^4^ or apoptotic. Emerging research has identified a role for regulatory T-cells in the development of radiation-induced pulmonary fibrosis^55^. However, it remains to be determined if IsoLG-activated T-cells impact this disease.

Eukaryotes use two major pathways to clear proteotoxic proteins: ubiquitination/proteasome-mediated degradation^56^ and autophagy^57^. Davies *et al*. have shown that IsoLG-modified proteins are poor substrates for proteasome-dependent degradation and that the proteasome itself can also be significantly impaired by IsoLG-modification^47^. Currently, it is not known if autophagy is impacted by IsoLG modification. We interpret our results to indicate that IsoLG-mediated cytotoxicity is not a consequence of targeting a specific prosurvival pathway, but is most likely due to the accumulation of toxic modified proteins that exceed a critical threshold.

Ionizing radiation is a well characterized prooxidant and was shown to induce IsoLG modification in cell culture and in a murine model of radiation-induced pulmonary fibrosis. It is well established that most chronic fibrotic diseases have in common a state of persistent injury^58^. Thus it was of particular interest to observe that ionizing radiation produces a state of chronic IsoLG protein-modification that correlates with chronic apoptosis and fibrosis^46^. IsoLG-modified proteins were also found to be a prominent feature of IPF. Our findings showing that collagen 1α1 is modified by IsoLGs in IPF patients and that adducted collagen impairs MMP1 degradation demonstrates the potential to induce a state of chronic injury and to impair resolution of established fibrosis. In summary, these results suggest that the excess oxidant burden associated with radiation-induced pulmonary fibrosis and with IPF drives the hitherto unrecognized pathogenic adduction of protein by IsoLG. Since IsoLG can be proteotoxic and injurious to cells, we expect that IsoLG is not only a marker of oxidant-stressed cells, but also augments lung injury and fibrosis.

## Materials and Methods

### Cell culture

Human microvascular endothelial cells (HMVECs), murine 3B11 endothelial cells, and murine alveolar type II MLE12 cells were obtained from ATCC and grown according to ATCC recommendations. Exponentially growing 3B11 cells were administered 5 Gy at room temperature using a Cesium-137 irradiator (2 Gy/min; JL Shepherd, Mark 1) and then incubated at 37 °C for 24 hours before harvesting. HMVECs were treated with 150 μM H_2_O_2_ in PBS for 1 hr at 37 °C, washed extensively, and incubated at 37 °C for 24 hours before harvesting.

### Mice

Congenic C57BL/6J mice with a targeted disruption of the *Nfe2l2* gene have been described previously^46^. Mice were X-irradiated between 7 and 10 months of age^46^. Mice were maintained under specific pathogen free conditions. All procedures performed on animals were approved by the Institutional Animal Care and Use Committee at Vanderbilt University and at the University of Texas MD Anderson Cancer Center, and complied with all state, federal, and NIH regulations.

Congenic C57BL/6J mice harboring a targeted disruption of the *Ncf1* gene have been described previously^59^. Mice were bred and maintained under specific pathogen free conditions at the animal care facility at Roswell Park Cancer Institute, Buffalo, NY. Mice were 8–15 weeks of age. All procedures performed on animals were approved by the Institutional Animal Care and Use Committee at Roswell Park Cancer Institute and complied with all state, federal, and NIH regulations.

### Immunohistochemistry (IHC)

Paraffin-embedded lung tissue (5 μm) were prepared at the Mouse Pathology Core Facility at Vanderbilt University. H&E stains were performed using standard protocols. Lung tissue sections were incubated with primary antibody overnight at 4 °C. Sections without primary antibody served as negative controls. Nitro-blue tetrazolium chloride (NBT) and 5-bromo-4-chloro-3′-indolyphosphate p-toluidine salt (BCIP, catalog no. 34070, Thermo Fisher Scientific) were used to produce localized visible staining. Slides were counterstained with methyl green. IHC stained IPF tissues were independently and blindly assessed for positive staining by Vanderbilt Idiopathic Pulmonary Fibrosis Center personnel.

### Immunofluorescence staining of mouse and human lung tissue

Mouse lungs were perfused with phosphate buffered saline, pH 7.2 (PBS) through the pulmonary artery and fixed with 10% phosphate-buffered formalin. After fixation lungs were processed and paraffin-embedded. Paraffin-embedded lung tissue was sectioned into 5 μm slices and mounted on glass slides.

The following primary antibodies were used: single chain ScFv anti-IsoLG antibody, (D11, 1:100 dilution, see (Davies *et al*.^28^ for details of preparation), Goat polyclonal anti-SPC antibody (catalog no. sc-7706, 1:100 dilution; Santa Cruz), Goat polyclonal anti-T1α Podoplanin antibody (catalog no. sc-23564, 1:100 dilution; Santa Cruz), Rabbit polyclonal anti-CC10 antibody (catalog no. sc-25555, 1: 100 dilution; Santa Cruz), Goat polyclonal anti-PECAM-1 antibody (catalog no. sc-1506, 1:100 dilution; Santa Cruz), Goat polyclonal anti-COL1A1 antibody (catalog no. sc-8784, 1:100 dilution; Santa Cruz). Of note, D11 ScFv recognizes peptides and proteins modified on the lysine residues by IsoLG isomers that arise from both the free radical and cyclooxygenase pathway and recognition is not dependent on amino acids adjacent to the modified lysine^28^. The D11 ScFv encodes the E-tag sequence used for detection. After primary antibody incubation, lung sections were washed in PBS. A secondary antibody raised against the E-tag epitope was then used to detect D11 binding. Anti-E-tag antibody was diluted in 10% BSA and sections were incubated 1 hr at RT. Rabbit polyclonal anti-E-tag antibody (catalog no. ab3397, 1:1,000 dilution; Abcam), was used for dual-stained with goat-raised primary antibodies; Goat polyclonal anti-E-tag antibody (catalog no. ab95868, 1:1,000 dilution; Abcam) was used for dual-stained with rabbit raised primary antibodies. After anti-E-tag antibody incubation sections were washed in PBS. Sections were then incubated with fluorescently tagged antibodies diluted in 10% BSA for 1 hr at RT. The following fluorescently tagged antibodies were used sequentially: Donkey Anti-Goat Alexa 647 (catalog no. A-21447, 1:1,000 dilution; Life Technologies) and Goat Anti-Rabbit Alexa 568 (catalog no. A-11011, 1:1,000 dilution; Life Technologies). After incubation with fluorescently tagged antibodies sections were then washed in PBS and mounted with ProLong Gold Antifade Mountant with DAPI (catalog no. P36931, Life Technologies) and sealed with glass coverslips. For specificity controls, primary antibody was not added.

### Immunofluorescence image acquisition

Confocal images were acquired using an Olympus FV-1000 inverted confocal microscope provided by the VUMC Cell Imaging Shared Resource. Z-stack images were taken at 0.45 um slice thickness using a 60×/1.45 Plan-Apochromat oil immersion objective lens. Wide-field immunofluorescence images were acquired using a Leica DM IRB inverted microscope equipped with a Nikon DXM1200C camera provided by the IMSD program at Vanderbilt.

### Immunofluorescence intensity quantification

Antibody-specific immunofluorescence intensity represents antibody-specific integrated density per field, as defined in and calculated by ImageJ (NIH), divided by percent DAPI staining per field in order to account for tissue cellularity. Background staining was quantified in the same manner and mean background staining was subtracted from antibody-specific immunofluorescence intensity using the threshold function on ImageJ. Relative intensity represents the quotient obtained by dividing experimental by control. We report mean relative intensity ± SD.

### Immuno-affinity isolation of IsoLG-modified proteins

Cells were lysed in 0.1% NP40-PBS, pH 7.2 and the lysate centrifuged at 12,000× g for 10 min. The supernatant was adjusted to 0.1%, NP40, 0.1% SDS, in PBS, pH 7.2. Proteins were extracted from the pellet using 500 mM NaCl-PBS, pH 7.2, brought to isotonic conditions (100 mM NaCl with 0.1%, NP40, 0.1% SDS, in PBS, pH 7.2), sonicated on ice, and then centrifuged at 12,000 × g for 10 min. The resulting supernatant was recovered.

Soluble protein was precleared using protein A/G beads. Ten ug D11 ScFv was used to isolate isoLG-modified proteins (16 hrs/4 °C). Anti-E-tag antibody conjugated agarose beads (catalog no. ab19368; Abcam) were used to capture D11 ScFv antibody. Beads were subsequently washed with 0.1% NP40, 0.1% SDS, in PBS pH7.2. Purified proteins were solubilized in 5× loading buffer and boiled for 7 mins, resolved approximately 3 cm on an SDS-PAGE gel, stained with coomassie blue and the entire lane excised for LC-MS/MS analysis.

As a control for D11 ScFv specificity the following reactions were performed: 500 uM 4-hydroxynonenal (HNE) was reacted with 5 mM lysine for 24 hrs at RT. Unreacted HNE was quenched using 50 mM Tris-HCl. Ten ug D11 ScFv was added to HNE-Lysine complex and incubated overnight at 4 °C prior to being used for immunoprecipitation of IsoLG-modified proteins.

### LC-MS/MS analysis of affinity purified proteins

D11 immunoaffinity purified proteins from sham and irradiated 3B11 cells were subjected to in-gel trypsin digestion and the resulting peptides analyzed by a 70 minute data dependent LC-MS/MS run. Briefly, peptides were auto--sampled onto a 200 mm by 0.1 mm (Jupiter 3 micron, 300A), self-packed analytical column coupled directly to an LTQ (ThermoFisher) linear ion trap mass spectrometer via a nanoelectrospray source and resolved using an aqueous to organic gradient. A series in which a full scan mass spectrum (MS) followed by 5 data-dependent tandem mass spectra (MS/MS) was collected throughout the run with dynamic exclusion enabled to minimize acquisition of redundant spectra. MS/MS spectra were searched via SEQUEST a mouse protein database (UniprotKB) that also contained reversed version for each of the entries. Spectral identifications were filtered and collated into spectral count numbers at the protein level using Scaffold (Proteome Software).

### Histone isolation from mouse lung tissue

Histone isolation was performed as described^35^.

### Human idiopathic pulmonary fibrosis and organ donor lung tissue

Collection, storage of samples and experimental protocols were carried out in accordance with relevant guidelines and regulations approved by the Vanderbilt University Institutional Review Boards (Vanderbilt IRB Protocol 9401). Written informed consent was obtained from all subjects. Human tissue sections were obtained from explanted IPF lungs at the time of transplant. Control tissue was obtained from lungs rejected for organ donation at Vanderbilt University. IPF diagnosis was confirmed by multidisciplinary review according to the 2011 ATS/ERS Consensus Guidelines^60^.

### Statistical Analysis

An unpaired t test or an analysis of variance was used for comparison between various groups. A *P* value less than 0.05 was considered as statistically significant.

## Additional Information

**How to cite this article**: Mont, S. *et al*. Accumulation of isolevuglandin-modified protein in normal and fibrotic lung. *Sci. Rep*. **6**, 24919; doi: 10.1038/srep24919 (2016).

## Supplementary Material

Supplementary Information

Supplementary Table S1

Supplementary Table S2
