# Supplementary material for: Accumulation of isolevuglandin-modified protein in normal and fibrotic lung
Source: Sci Rep. 2016 Apr 27;6:24919. doi: 10.1038/srep24919 (PMC4847119; doi:10.1038/srep24919)
Supplement: Supplementary Information [file srep24919-s1.pdf]

Accumulation of isolevuglandin-modified protein in normal and fibrotic lung

Stacey Mont[1,2], Sean S. Davies[3], L. Jackson Roberts 2nd[3], Raymond L. Mernaugh[4], W. Hayes McDonald[4,5], Brahm H Segal[6], William Zackert[3], Jonathan A Kropski[7], Timothy S. Blackwell[7], Konjeti R. Sekhar[2], James J. Galligan[4], Pierre P. Massion[7], Lawrence J. Marnett[4,8], Elizabeth L. Travis[9], and Michael L. Freeman[2*]

[1]Department of Cancer Biology, [2]Department of Radiation Oncology, [3]Division of Clinical Pharmacology, Department of Pharmacology, [4]Department of Biochemistry, [5]Proteomics Laboratory and Mass Spectrometry Research Center, [7]Division of Pulmonary & Critical Care, Department of Medicine, [8]A.B. Hancock Jr. Memorial Laboratory for Cancer Research, Vanderbilt Institute of Chemical Biology, Vanderbilt-Ingram Cancer Center, Vanderbilt University Medical Center, Nashville, TN 37240, USA; [6]Department of Medicine, Department of Immunology, Roswell Park Cancer Institute, and University at Buffalo Jacobs School of Medicine and Biomedical Sciences, Buffalo, NY, 14263; [9]Department of Experimental Radiation Oncology, Division of Radiation Oncology, The University of Texas MD Anderson Cancer Center, Houston, TX 77230

*Correspondence should be addressed to MLF (email: michael.freeman@vanderbilt.edu)

Supplementary Information:

Materials and Methods

Quantitative real time PCR. Total RNA was extracted from needle-picked human lung tissues using Arcturus PicoPure RNA isolation kit (Applied Biosystems). RNA was quantified using nano-drop methodology with Biotek Synergy HT. RT-PCR was conducted in triplicate using an iScript One-Step kit with SYBR Green (BioRad). Primers used for RT-PCR were *NFE2L2*-F: 5'-AGTGGATCTGCCAACTACTC-3'; *NFE2L2*-R: 5'-CATCTACAAACGGGAATGTCTG-3'; Actin-F: 5'-TCACCCACACTGTGCCCATCTACGA-3'; Actin-R: 5'-CAGCGGAACCGCTCATTGCCAATGG-3'. A PCR standard curve was generated using pcDNA3/NFE2L2 expression construct using iTaq Universal SYBR Green Supermix kit (BioRad).

Measurement of apoptosis. Percent of apoptosis was measured using the Annexin V-fluorescein isothiocyanate apoptosis detection kit I (Pharmingen) with flow cytometry according to the manufacturer's directions.

MMP-1 degradation assay with Collagen1Alpha1. Purified human recombinant Col1α1 was incubated with purified IsoLG at 37°C/1hr, after which unreacted IsoLG was quenched. MMP1 was the added to the reaction and incubated at 37°C/0.5hr, pH = 7.0. Col1α1 degradation was analyzed by 1D SDS PAGE, Coomassie Blue staining.

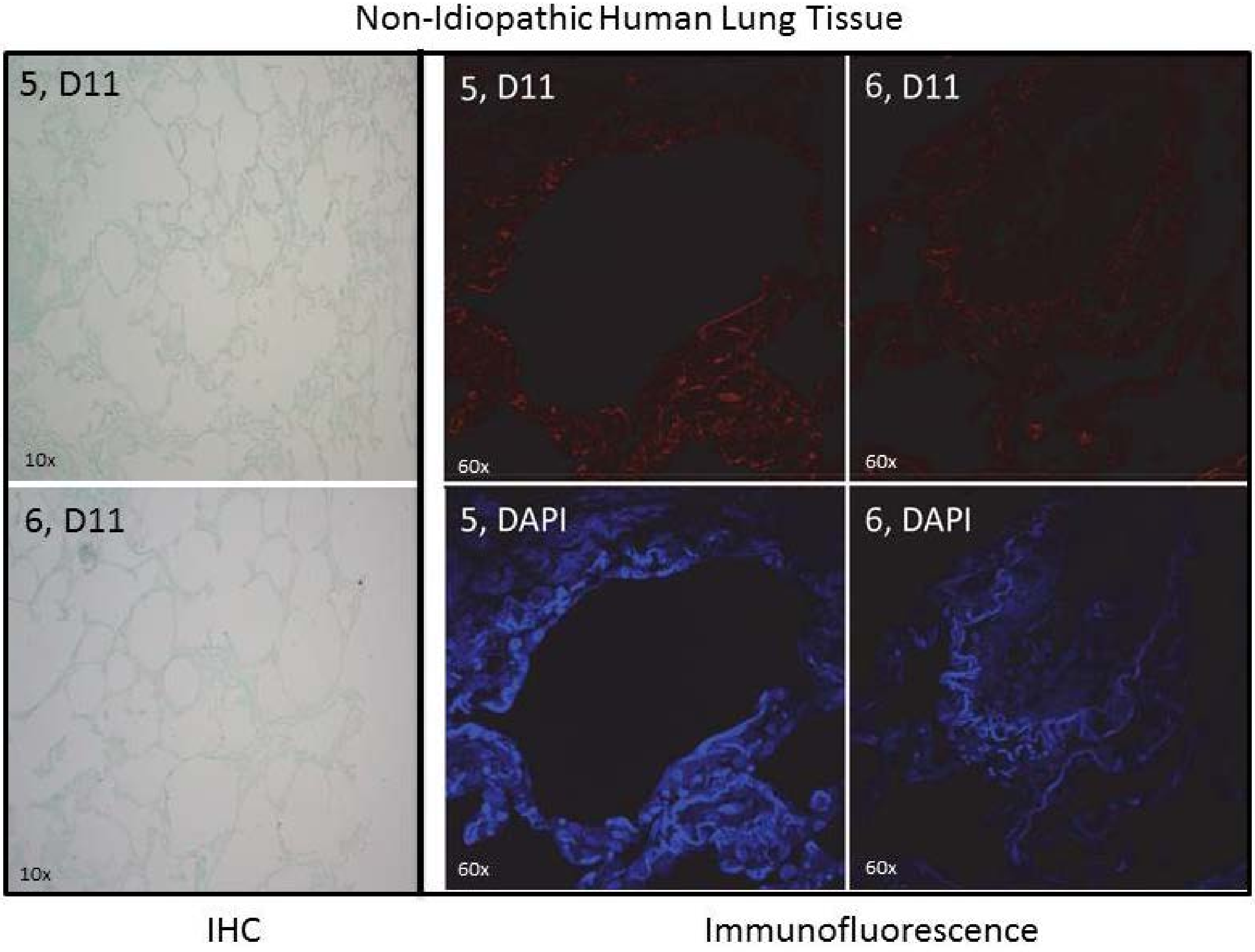

Legend for Supplementary Fig 1: Human lung tissue sections from organ donors 5 and 6 underwent IHC staining with the D11 antibody, counterstained with methyl green and imaged using wide field microscopy or were immunostained with D11 (Red), counterstained with DAPI (blue) and imaged by confocal microscopy.

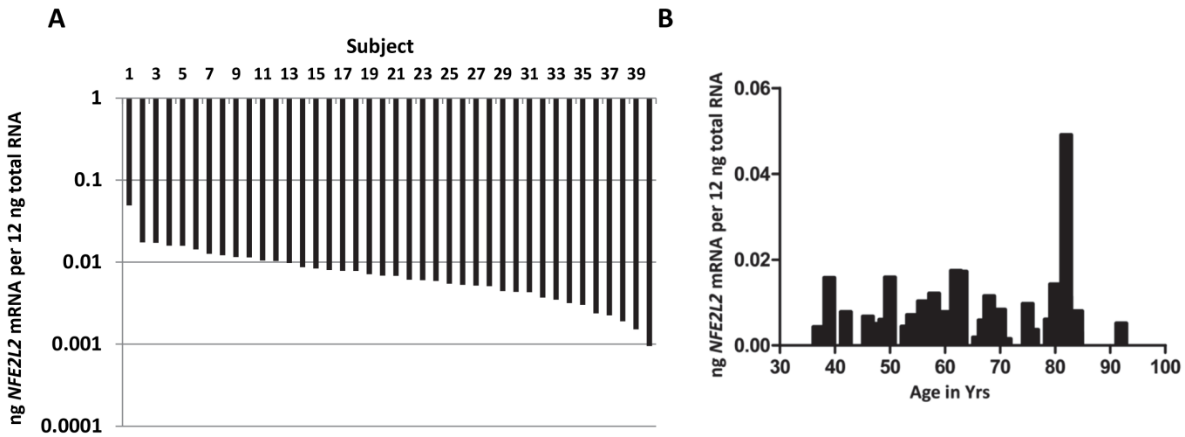

Legend for Supplementary Fig 2: De-identified fine-needle bronchial biopsies were obtained from 40 human individuals. (A) Waterfall plot of *NFE2L2* mRNA, quantified in triplicate relative to actin mRNA in non-cancerous bronchial tissue by qRT-PCR; (B) Expression of *NFE2L2* mRNA as a function of age.

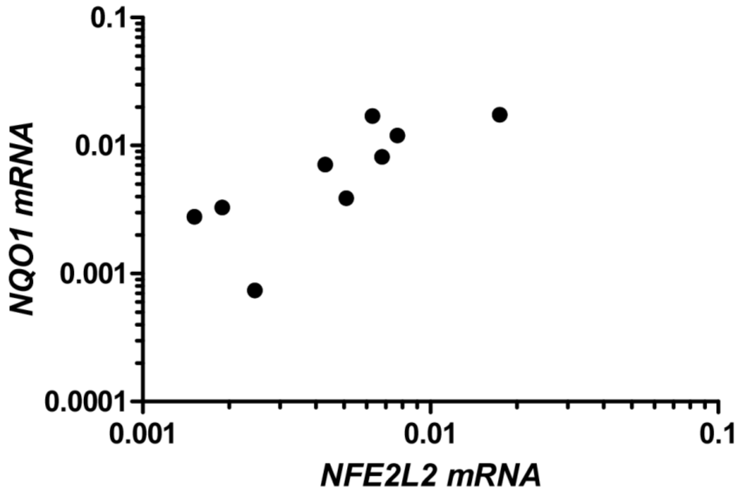

Legend for Supplementary Fig 3: The relationship between expression of *NFE2L2* mRNA and expression of *NQO1* mRNA in human non-cancerous pulmonary tissue, measured by qRT-PCR.

Supplementary Fig 4A

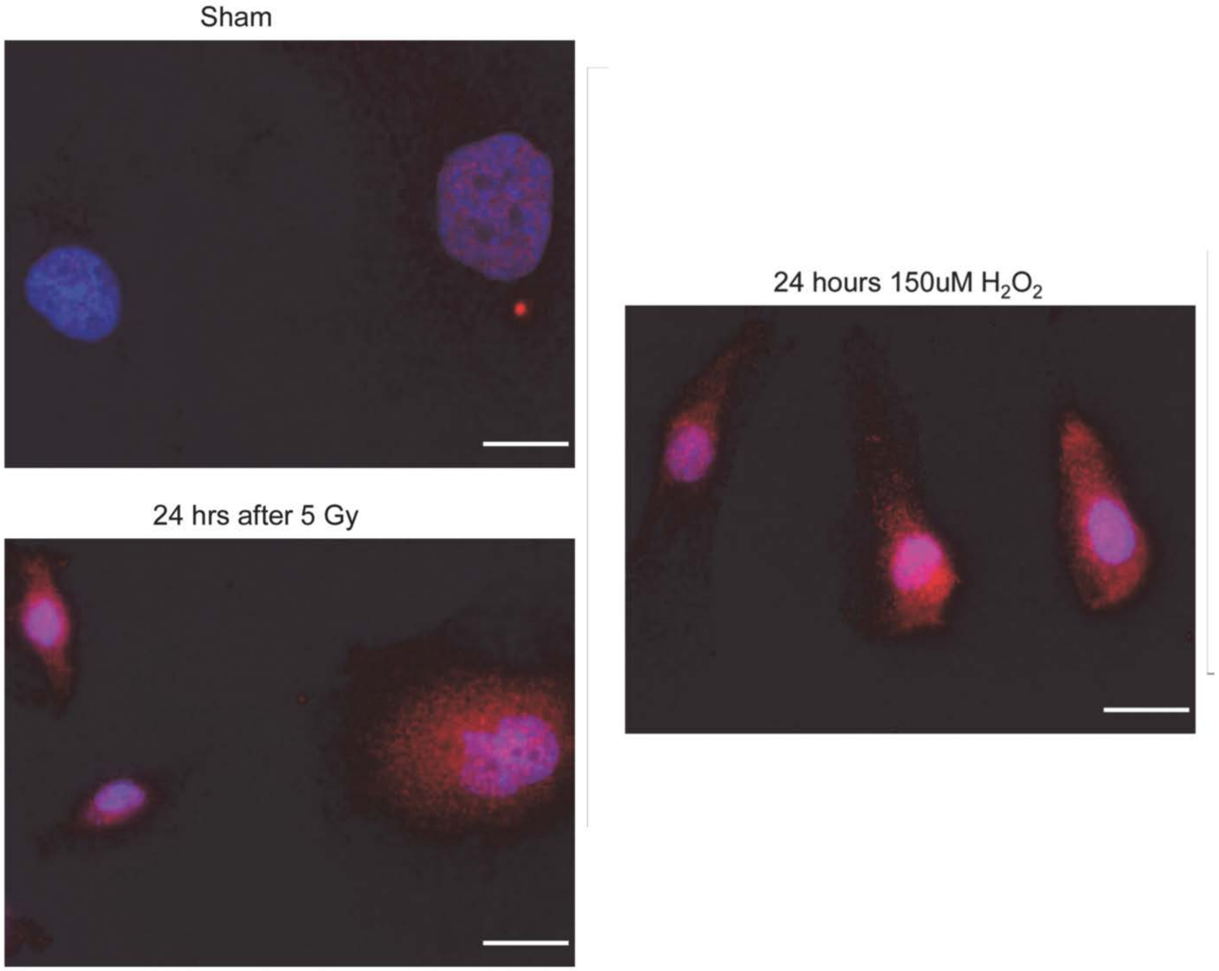

Supplementary Fig 4B

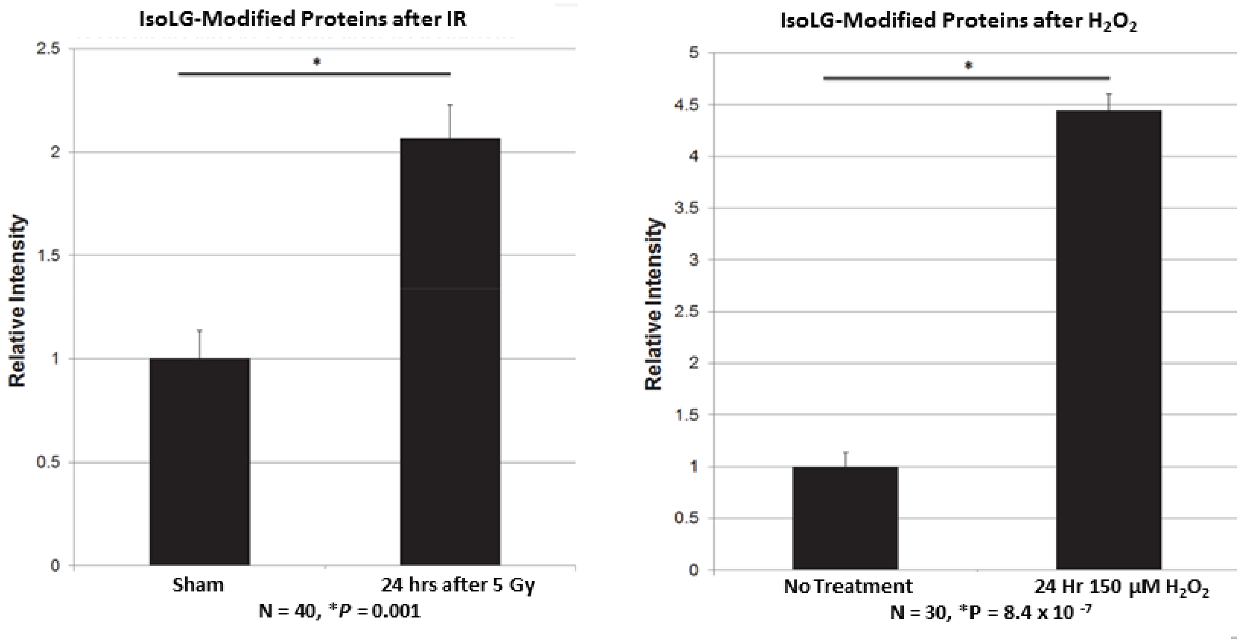

Legend for Supplementary Fig 4: A) Ionizing radiation and hydrogen peroxide induce formation of IsoLG-modified proteins. Human microvascular endothelial cells were stained for IsoLG-protein-adducts (Red) before and 24 hrs after administration of 5 Gy of γ-rays or 150uM hydrogen peroxide. **B)** Relative D11 staining intensity normalized to no treatment controls. Staining was measured at 60x magnification by wide-field microscopy and quantified by NIS Elements AR (Nikon).

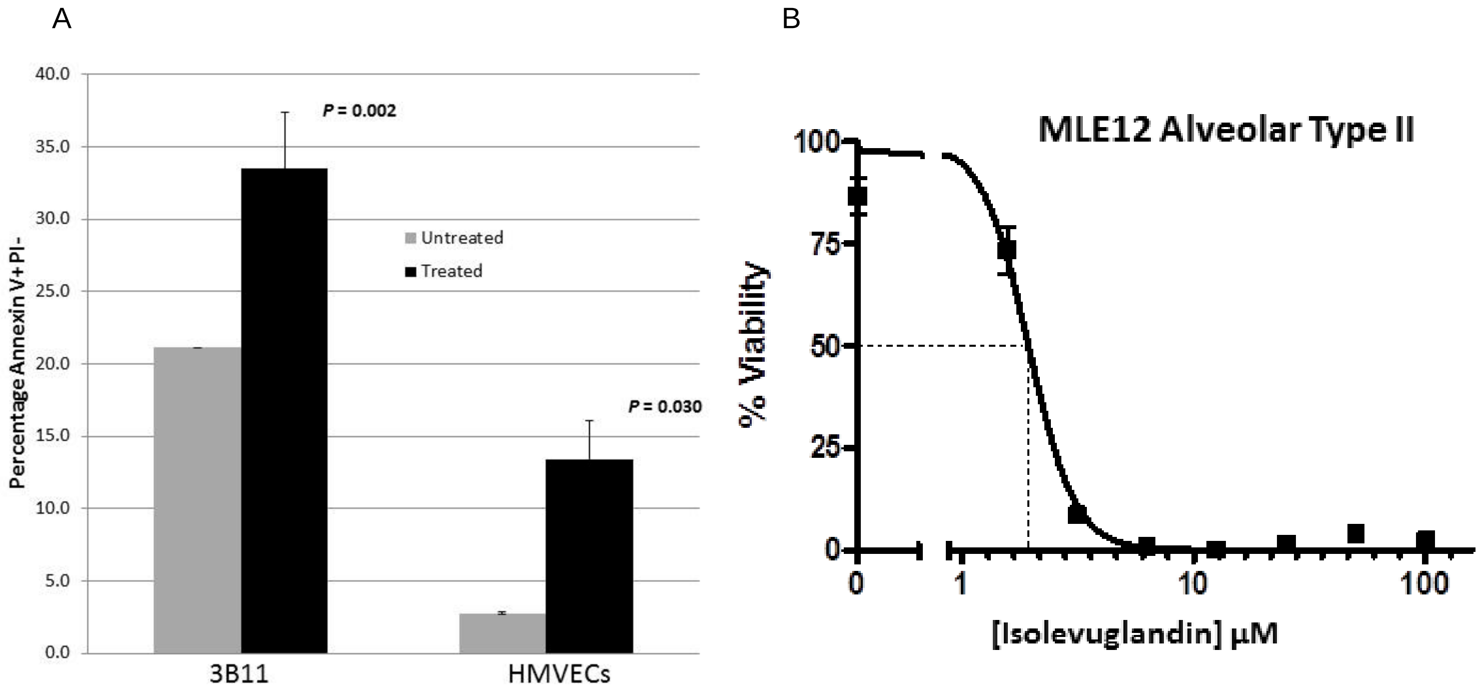

Legend for Supplementary Fig 5: IsoLGs are cytotoxic**.** A) Apoptosis of 3B11 and HMVECs exposed to 1µM 15-E_2-IsoLG for 1 hr. Sixteen hrs later apoptosis was measured by Annexin V+ PI- stained cells (mean ±SD, N = 3). B) Loss of viability in MLE12 cells exposed to various concentrations of 15-E_2-IsoLG for 1 hr. Sixteen hrs later an MTT assay was used to quantify viability (mean ±SD, N = 4). Standard deviations are shown if larger than symbols.

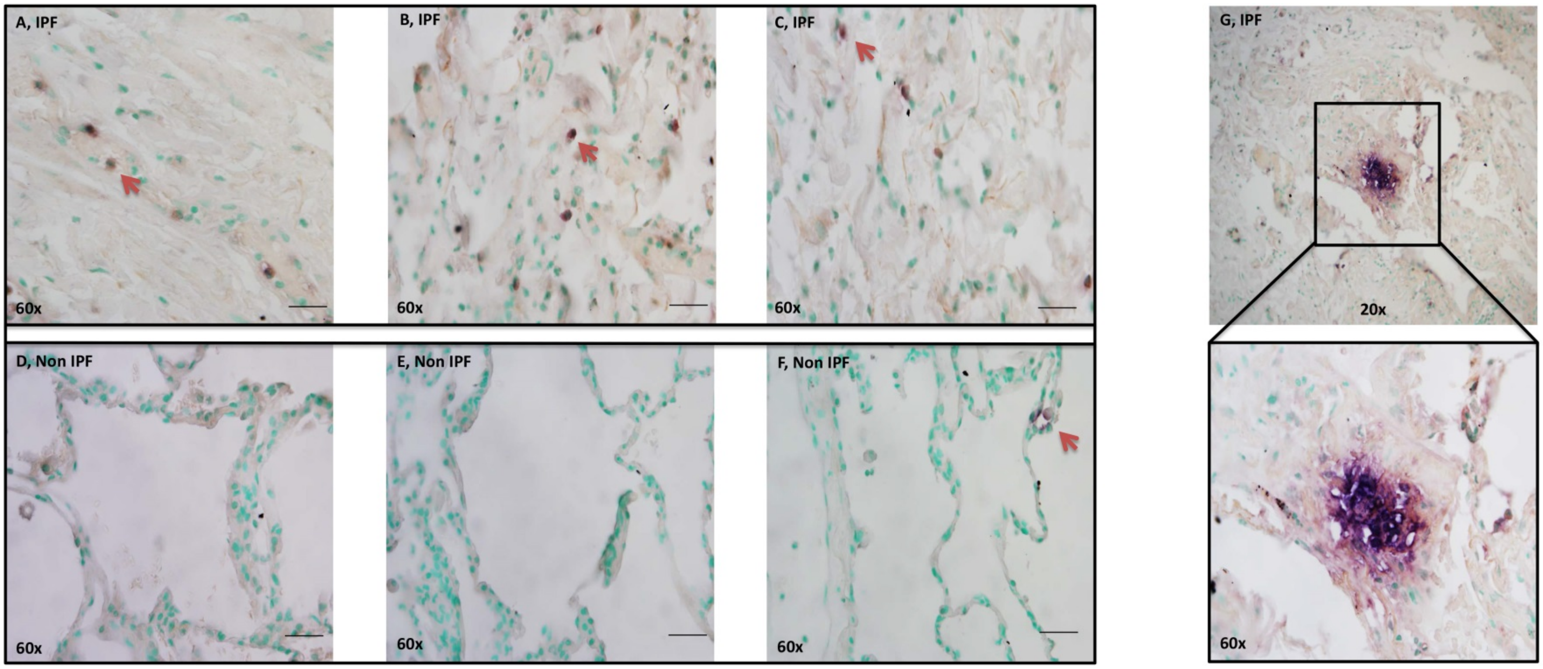

Legend for Supplementary Fig 6: Human lung tissue sections obtained from IPF patients (panels A-C & G) or non-IPF organ donors (panels D-F). FFPE sections were subjected to IHC staining with D11 and counter stained with methyl green. Sections were imaged using wide field microscopy. Black bar represents 30 µM. Red arrowhead denotes positively stained cells in panels A- F.

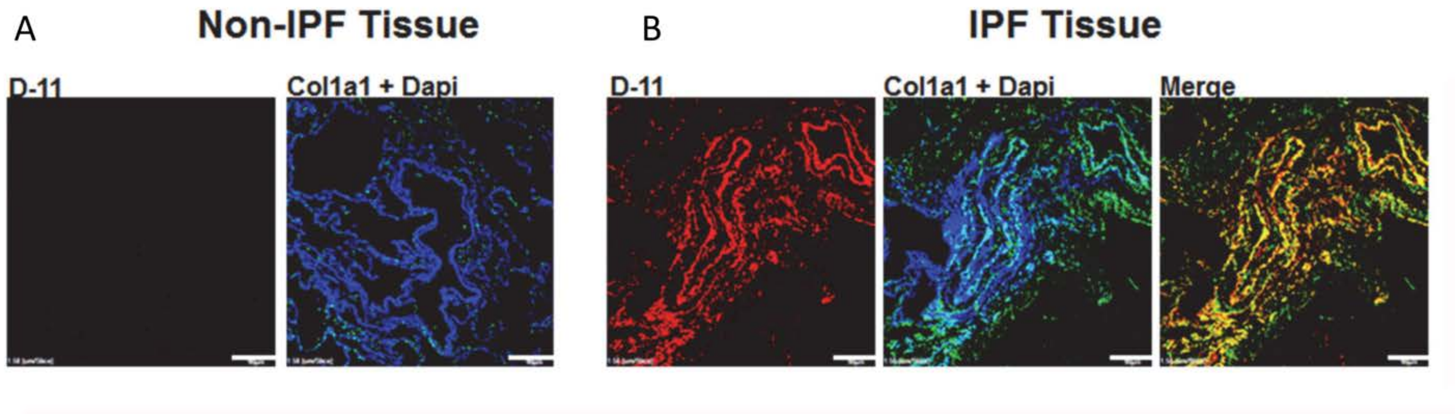

Legend for Supplementary Fig 7: IsoLG-modified proteins are present in human idiopathic pulmonary fibrotic tissue and colocalize with collagen. Human lung tissue sections from an organ donor (A) or from a subject with IPF (B) were stained with D11 (Red) and collagen type 1 alpha 1 (Alexa 647, green false color) and imaged by confocal microscopy. 20x magnification, N= 150 fields. The white bar represents 30 µm.

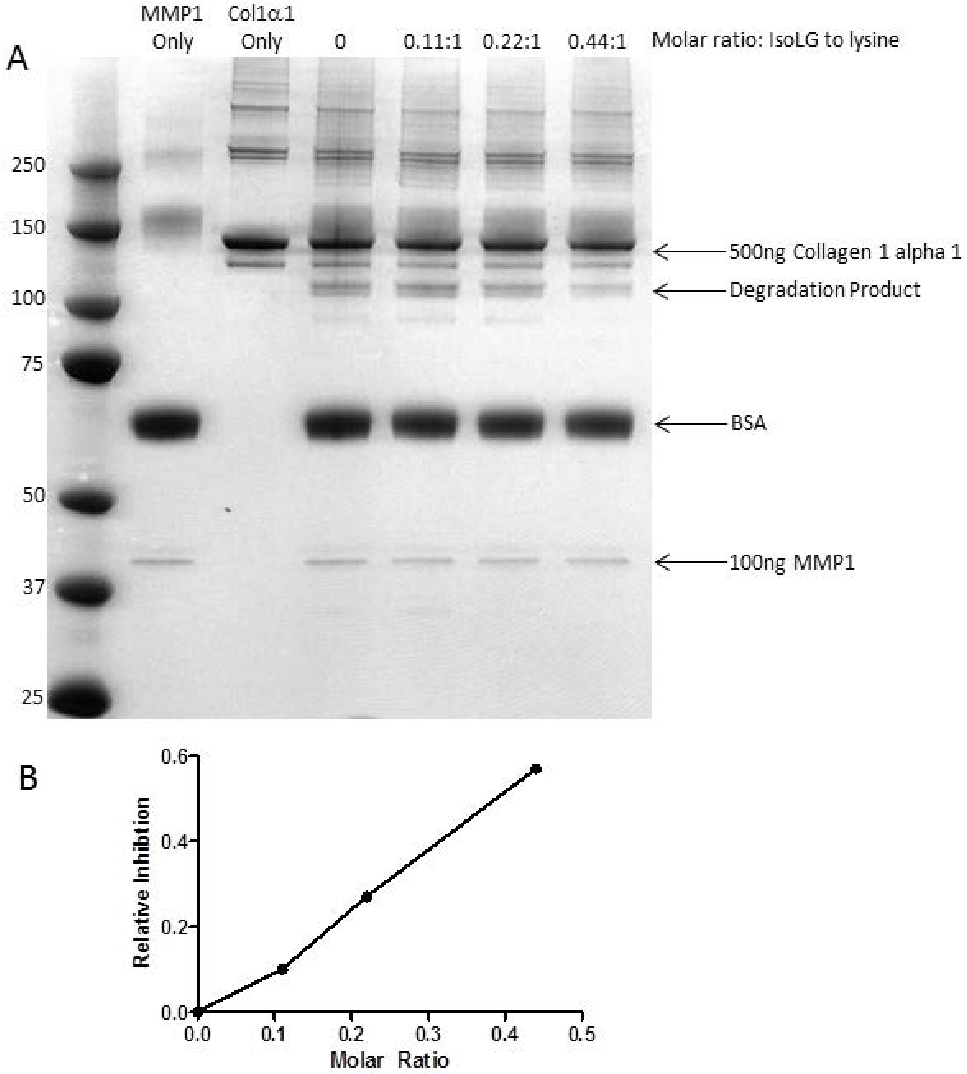

Legend for Supplementary Fig 8: MMP1 mediated degradation of collagen 1α1 (Col1α1). A) Purified human recombinant Col1α1 was incubated with the indicated molar ratios of purified IsoLG at 37°C/1hr, after which unreacted IsoLG was quenched. MMP1 was the added to the reaction and incubated at 37°C/0.5hr, pH = 7.0. Col1α1 degradation was analyzed by 1D SDS PAGE, Coomassie Blue staining. B) Intensity of Col1α1 degradation product was quantified and is shown as relative inhibition.

Supplementary Table 1
Patient Characteristics

Subject Number	Age	Gender	Diagnosis	Race/Ethicity	Tobacco Use	Pack Years	FVC%
1	67	Female	IPF	Caucasian	Yes	90	68
2	67	Male	IPF	Caucasian	Yes	42	82
3	61	Male	IPF	Caucasian	Yes	15	51
4	59	Female	Control	Caucasian	Yes	Unknown	Unknown
5	52	Female	Control	Caucasian	Unknown	Unknown	Unknown
6	32	Female	Control	Caucasian	No	NA	Unknown

Supplementary Table 2
Patient Characteristics

Subject Number	Age	Gender	Smoking Status	Histologic Type	Histology Description	Cancer History	Sample Type
1	68	Male	Never Smoker	Normal	respiratory epithelial hyperplasia	Colon	Bronchial Biopsy
2	58	Female	Ex-Smoker	Normal	Necrotizing Granulomatous, consistent with Blastomyces	No Prior	Bronchial biopsy
3	68	Male	Never Smoker	Normal	Necrotizing Granulomas, histiocytes, and fungal organisms	No Prior	Bronchial Biopsy
4	56	Male	Never Smoker	Normal	non-caseating granulomatous inflammation, Sarco	No Prior	Bronchial Biopsy
5	54	Female	Never Smoker	Normal		No Prior	Bronchial Biopsy
6	80	Male	Ex-smoker	Normal		No Prior	Bronchial biopsy
7	55	Female	Never Smoker	Normal	Nectrotizing Granuloma, Consistent with Histoplasmosis	No Prior	Bronchial Biopsy
8	42	Female	Ex-Smoker	Normal		No Prior	Bronchial Biopsy
9	56	Female	Never Smoker	Normal	Organizing Pneumonia	No Prior	Bronchial Biopsy
10	76	Male	Ex-Smoker	Normal	Benign solitary fibrous tumor	Melanoma Skin	Bronchial Biopsy
11	54	Female	Never Smoker	Normal	Necrotizing granulomas	No Prior	Bronchial Biopsy
12	53	Male	Ex-Smoker	Normal	Caseating Granuloma	Melanoma Skin	Bronchial Biopsy
13	82	Male	Ex-smoker	Normal	Anthracosis	No Prior	Bronchial biopsy
14	84	Male	Ex-Smoker	Normal	COPD	No Prior	Bronchial Biopsy
15	49	Female	Never Smoker	Normal	nectorizing granulomata and histoplasma	No Prior	Bronchial Biopsy
16	81	Female	Never Smoker	Normal	parabronchiolar fibrosis	No Prior	Bronchial Biopsy
17	67	Female	Ex-Smoker	Normal	Interstitial fibrosis, scarring & chronic inflammation	No Prior	Bronchial Biopsy
18	37	Male	Never Smoker	Normal	Sarcoid	No Prior	Bronchial Biopsy
19	46	Female	Ex-Smoker	Normal	Non-Necrotizing Granulomatous Inflammation	No Prior	Bronchial Biopsy
20	63	Female	Never Smoker	Normal	Necrotizing granulomas, Recurrent Breast Cancer, resected	Breast	Bronchial Biopsy
21	68	Male	Ex-smoker	Normal	COPD	No Prior	Bronchial biopsy
22	82	Female	Never Smoker	Normal	Caseating Granuloma, Acid-Fast Bacilli; Emphysema	Head and Neck	Bronchial Biopsy
23	48	Male	Ex-Smoker	Normal	IA NSCLC in '08, negative for cancer in '09	No Prior	Bronchial Biopsy
24	66	Male	Never Smoker	Normal	Necrotizing granulomatous inflammation, histoplasmosis	No Prior	Bronchial Biopsy
25	84	Male	Ex-Smoker	Normal	Necrotizing granuloma	NSCLC	Bronchial Biopsy
26	75	Male	Never Smoker	Normal		Prostate	Bronchial Biopsy
27	67	Male	Ex-Smoker	Normal	Blastomycosis, necrotizing granulomatous inflammation	Skin x3	Bronchial Biopsy
28	62	Female	Ex-smoker	Normal		Liver	Bronchial biopsy
29	92	Male	Ex-Smoker	Normal	COPD, Chronic atrophic gastritis marked intestinal metplasia, Interstitial lung	No Prior	Bronchial Biopsy
30	39	Female	Never smoked	Normal	Necrotizing Granuloma, consistent with Histoplasma Species	No Prior	Bronchial biopsy
31	81	Female	Never smoked	Normal	parabronchiolar fibrosis	No Prior	Bronchial biopsy
32	71	Male	Ex-Smoker	Normal		NSCLC	Bronchial Biopsy
33	79	Male	Ex-Smoker	Normal	Benign enlarged reactive adenopathy	Skin x4	Bronchial Biopsy
34	72	Male	Ex-Smoker	Normal	mediastinal lymphadenopathy	No Prior	Bronchial Biopsy
35	60	Female	Never Smoker	Normal	features are suggestive of an autoimmune disease	Skin, Gynecological	Bronchial Biopsy
36	46	Female	Never Smoker	Normal	Interstitial Fibrosis	No Prior	Bronchial Biopsy
37	70	Male	Ex-Smoker	Normal		Lung	Bronchial Biopsy
38	50	Male	Ex-smoker	Normal		No Prior	Bronchial biopsy
39	63	Male	Never smoked	Normal		No Prior	Bronchial biopsy
40	54	Male	Ex-Smoker	Normal		No Prior	Bronchial Biopsy
